# Antiviral activity of nitazoxanide against pseudorabies virus infection *in vitro*

**DOI:** 10.3389/fvets.2025.1623545

**Published:** 2025-06-16

**Authors:** Lei Tan, Pei Zhu, Zhaori Getu, Xi Yang, Shiling Zheng, Yuqing Duan, Jinping Wang, Yi Zhou, Yuyang Hu, Yijing Wang, Yuying Yang, Mengting Zuo, Jun Yao

**Affiliations:** ^1^College of Animal Science and Technology, Yangtze University, Jingzhou, Hubei, China; ^2^Yunnan Tropical and Subtropical Animal Virus Diseases Laboratory, Yunnan Animal Science and Veterinary Institute, Kunming, Yunnan, China; ^3^Chifeng Academy of Agricultural and Animal Husbandry, Chifeng, Inner Mongolia, China; ^4^Hunan Provincial Key Laboratory of the TCM Agricultural Biogenomics, Changsha Medical University, Changsha, Hunan, China

**Keywords:** nitazoxanide, pseudorabies virus, antiviral activity, viral replication phase, RNA-Seq

## Abstract

Pseudorabies (PR), an infectious disease caused by pseudorabies virus (PRV), has been responsible for substantial economic losses within the global swine industry. However, effective control measures and vaccines against PRV remain limited, thereby underscoring the necessity for the development of innovative antiviral agents targeting PRV. Nitazoxanide (NTZ) is an FDA-approved anthelminthic drug, has shown efficacy in inhibiting a variety of viral infections. This study aims to evaluate the antiviral properties of NTZ against PRV infection *in vitro*. The findings demonstrated that NTZ treatment significantly inhibited PRV infection in a dose-dependent manner in both PK15 and Vero cell lines, with the primary inhibitory effect occurring during the viral replication phase, rather than during the attachment, entry, or release phases of the virus. Subsequent RNA-Seq analysis revealed that cellular signaling pathways related to oxidative stress were implicated in the antiviral efficacy of NTZ against PRV infection. In conclusion, our findings implicate that NTZ effectively suppress PRV infection *in vitro*, suggesting its potential as a promising antiviral candidate for the clinical interventions in *Alphaherpesvirinae* infections.

## 1 Introduction

Pseudorabies virus (PRV), also known as Aujeszky’s disease virus, is an enveloped, double-stranded, linear DNA virus that belongs to the family *Orthoherpesviridae* and the subfamily *Alphaherpesvirinae*, which consists of both PRV and Herpse simplex virus 1 (HSV-1) (1–3). PRV has the capacity to infect a diverse range of mammals, including domestic and wild swine, ruminants (such as sheep, cow, cattle, and goats), as well as canine (e.g., dogs, wolves, and foxes) (4, 5). In swine, PRV infection manifests clinically through reproductive disorders in sows and central nervous system disorders in piglets. In non-natural hosts, PRV infection can be fatal, presenting with severe pruritus and neurological symptoms (6). Additionally, over 30 human cases of PRV infection have been documented in China, underscoring the potential risk of zoonotic transmission from pigs to humans (7, 8). Nevertheless, the availability of effective therapeutic options for managing PRV infections remains limited.

PRV has been widely prevalent in pig populations across various regions, including Asia (notably in China, South Korea, and Japan) (4, 9, 10), Europe (specifically in Italy, Greece, and France) (5, 11, 12), and the United States (13). The genetic characteristics of PRV strains found globally indicate that they can be categorized into two distinct genotypes: genotype I and genotype II (14). Genotype I PRV strains are predominantly found in countries within Europe and America, as well as in China (6). However, the majority of PRV strains circulating in China are classified as genotype II, which can be further divided into two subgroups: variant and classical PRV strains (6). Specially, the variant PRV strains that have emerged since 2011 exhibit increased pathogenicity compared to classical PRV strains, and these variants are now considered as the primary PRV strains prevalent in China (15, 16).

Nitazoxanide (NTZ) is a well-established anti-parasitic agent that has been extensively used for the treatment and management of *Cryptosporidium* infections (17). Recent studies have revealed that NTZ possesses a range of additional biological activities, such as anti-cancer (18), antibacterial (19), antiviral (20), and anti-inflammatory properties (21). Notably, NTZ has demonstrated the ability to inhibit the infections caused by various swine viruses, including the Porcine reproductive and respiratory syndrome virus (PRRSV) (22), Influenza A virus (23), Porcine epidemic diarrhea virus (24), and Japanese encephalitis virus (JEV) (25). For example, NTZ treatment has shown significant antiviral efficacy in reducing the proliferation and transmission of PRRSV in a swine model, indicating its potential as a promising therapeutic agent for the management of PRRSV infections in clinical contexts (22). However, the functions of NTZ in the context of PRV infection and its underlying mechanisms remain to be elucidated.

In the present study, we investigated the antiviral activities of NTZ against PRV infection *in vivo*. Our findings showed that NTZ treatment markedly suppressed PRV infection in both PK15 and Vero cell lines, primarily by disrupting the viral replication phase. Subsequent transcriptomic analysis indicated that the oxidative stress pathways were involved in the antiviral efficacy of NTZ against PRV infection *in vitro*. In conclusion, our results suggested that NTZ may serve as a promising novel antiviral candidate for the treatment and management of PRV.

## 2 Materials and methods

### 2.1 Cells, viruses, chemicals, and antibodies

Vero cells and PK-15 cells were cultivated in our laboratory using Dulbecco’s Modified Eagle Medium (DMEM) supplemented with 10% fetal bovine serum (FBS) and 1% penicillin–streptomycin at 37°C in an atmosphere containing 5% CO_2._ The variant (PRV-HuN-LD, PRV-YuN-KM) and classical (PRV-HuN-XT) PRV, and HSV-1 strains were isolated and preserved in our laboratory, these strains are utilized to assess the extensive antiviral efficiency of NTZ against infections caused by *Alphaherpesvirinae in vitro*.

**Figure 1:**
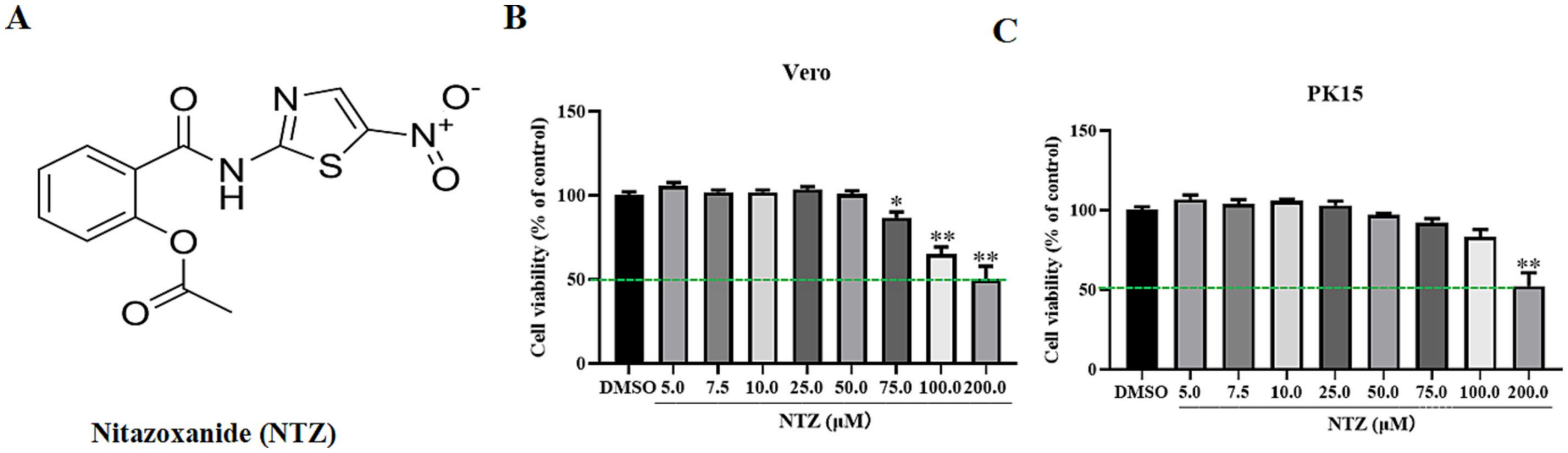
The chemical structure and cytotoxicity of NTZ on PK15 and Vero cell lines. **(A)** The chemical structure of NTZ. **(B,C)** PK15 or Vero cells were incubated with NTZ (0–200 μM) for 48 h, the cell viability was assessed using a CCK-8 method (%).

Antibody against PRV gC was kindly provided by Doctor Juan Li in Yunnan Sino-Science Gene Technology CO. ltd (Kunming, China). Antibody against *β*-actin (ab8226) were purchased from the Abcam (United States). NTZ (Figure 1A) was purchased from MedChemExpress and dissolved in dimethyl sulfoxide (DMSO) to achieve a final concentration of 250 mM.

### 2.2 Cytotoxicity assay

PK15 or Vero cells were cultured in 96-well plates and treated with DMEM medium supplemented with 10% FBS alongside varying concentrations of NTZ (5.0, 7.5, 10.0, 25.0, 50.0, 100.0, and 200.0 μM) or DMSO for a duration of 48 h. The assessment of cell viability was conducted using the CCK-8 assay, with comprehensive procedural details provided in Tan’s study (16).

### 2.3 RNA extraction and RT-qPCR analysis

The total RNA genome of the cells was extracted using a commercial SteadyPure Universal RNA Extraction Kit (Accurate Biology, Hunan, China). Nearly 1.0 μg of RNA was used to synthesize complementary DNA (cDNA) with the PrimerScript RT Master Mix (Accurate Biology, Hunan, China). RT-qPCR assay was performed to analyze the relative transcript levels of the targeted genes using the 2^-△△CT^ method as described previously (26), using GAPDH as an internal reference gene. These specific primers were synthesized by Qingke Biotechnology Co., Ltd. (Beijing, China), and their details were presented in Supplementary Table 1.

### 2.4 Western blotting analysis

The relative protein expression levels of PRV-gC were assessed using western blotting assay. In brief, total cellular proteins were extracted using the NP40 lysis buffer containing protease inhibitors. Equal amounts of protein specimens were separated by 10% SDS-PAGE and transferred onto PVDF membranes. The membranes were then blocked with 5% defatted milk powder overnight at 4°C, and incubated with specific primary antibodies (PRV-gC and *β*-actin) for 4 h at 37°C. Subsequently, the membranes were washed five times with PBST, and incubated with DyLight 800 goat anti-mouse IgG antibodies at 37°C for 1 h. Finally, the target proteins were visualized using an enhanced chemiluminescence substrate (ECL) kit (New Cell & Molecular Biotech, Suzhou, China).

### 2.5 Indirect immunofluorescence assay

PK15 cells seeded in a 12-well plate were incubated with 4% paraformaldehyde for 15 min at 37°C, followed by a 10-min incubation with 0.1% Triton X-100. After washing three times with PBS, cells were blocked with PBS supplemented with 3% BSA at 37°C for 2 h and and then incubated with mouse anti-PRV gC monoclonal antibody (1:1000 dilution) at 4°C overnight. The cells were washed five times with PBS and subsequently incubated with the FITC-labeled goat anti-mouse secondary antibody (1:3000 dilution; KPL, United States for 45 min in a dark box. Finally, the cells were washed with PBS and subjected to DAPI counterstaining, the green (specific to the PRV-gC protein) and blue (specific to the nucleus) signals were examined by fluorescence microscopy.

### 2.6 Effect of NTZ treatment on PRV infection in Vero and PK15 cells

Cells cultured in 6-well plates were pre-treated with varying concentrations of NTZ (0, 12.5, 25.0, and 50.0 μM) for 2 h. Subsequently, the cells were infected with PRV strain at a multiplicity of infection (MOI) of 0.1 for 1 h, while maintaining the respective concentrations of NTZ. Cells were washed three times with PBS to remove the supernatants, and then cultured in DMEM medium supplemented with 10% FBS and the corresponding concentrations of NTZ. At 24 h post-infection (hpi), cells were harvested to assess PRV replication via western blotting, IFA, and RT-qPCR analysis. At the same time, the supernatants were collected to assess viral titer using TCID_50_, as previously described (17).

### 2.7 Analysis of temporal dynamics in the life cycle of PRV infection influenced by NTZ

**Figure 2:**
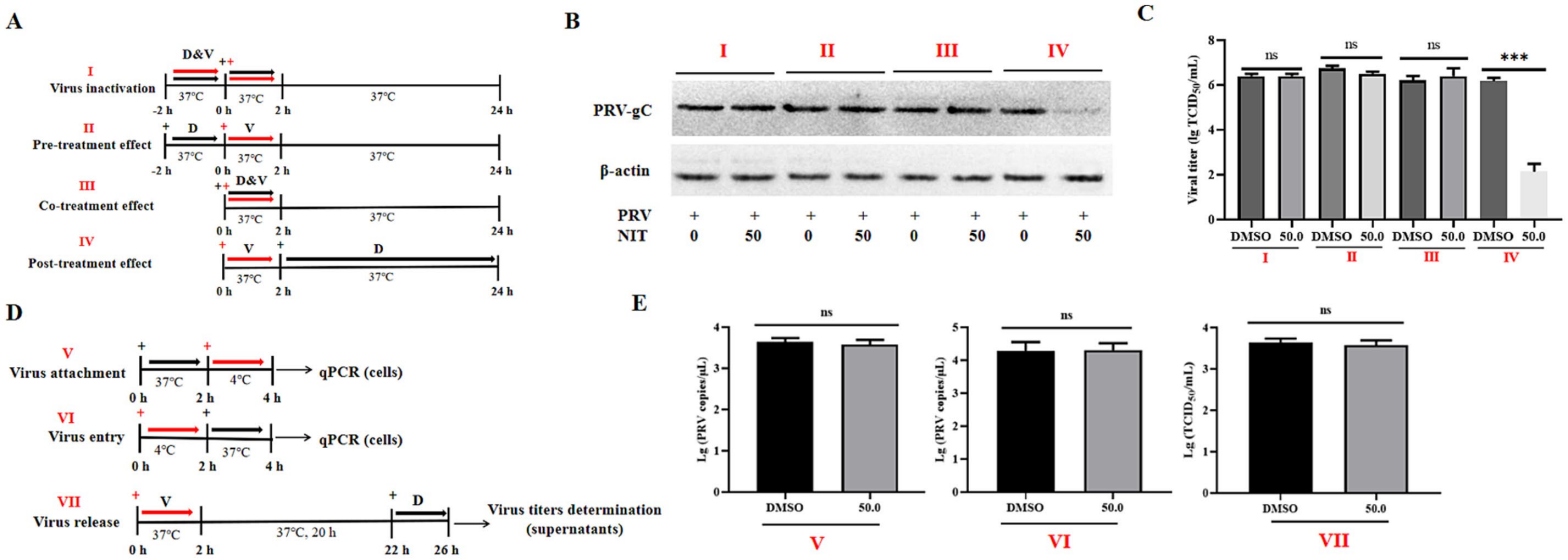
Effects of NTZ on different phases of PRV infection in Vero cells. **(A)** A schematic representation of virus inactivation (I), pre-treatment (II), co-treatment (III), and post-treatment (IV), then cells and supernatants were harvested for western blotting **(B)** and viral titer determination **(C)**. **(D,E)** A schematic representation and results of virus attachment (V), virus entry (VI), and virus release assays (VII).

To assess which phase of the life cycle of PRV has been influenced by NTZ, Vero cells were seeded in 12-well plates and treated with NTZ under different conditions, which included viral inactivation, pre-treatment, co-treatment, and post-treatment (Figure 2A). After these treatments, cells were collected for western blotting analysis at 24 hpi. Meanwhile, the supernatants were obtained to evaluate the viral titers, as previously described (17).

### 2.8 Assays for virus attachment, entry, and release

Using the PRV-HuN-LD strain as a model, Vero cells were cultured in 6-well plates and categorized into three distinct groups to examine the antiviral mechanisms of NTZ against PRV infection *in vitro*, as illustrated in Figure 2D.

Viral Attachment Assay: Vero cells were pre-treated with either DMEM or DMEM supplemented with NTZ (50.0 μM) for 2 h. Subsequently, 10^6^ TCID50 of PRV-HuN-LD was added to the cell plates and incubated for 2 h at 4°C to facilitate viral attachment. After removing the supernatants, the cells were washed three times with cold PBS. Total DNA was extracted from the cells using a commercial DNA/RNA extraction kit (Takara, Dalian, China). The number of PRV DNA copies was determined using qPCR, as previously described (27).

Viral Entry Assay: Vero cells were incubated with 10^6^ TCID_50_ of PRV-HuN-LD for 2 h at 4°C to facilitate virus attachment. After removing the supernatants, the cells were treated with DMEM medium containing 10% FBS, with or without NTZ (50.0 μM) for 2 h at 37°C to allow for virus entry. Total DNA was extracted from the cells using a commercial DNA/RNA extraction kit (Takara, Dalian, China). The number of PRV DNA copies was determined using qPCR, as previously described (27).

Viral release assay: Vero cells were infected with 10^3^ TCID_50_ of PRV-HuN-LD for 2 h at 37°C to permit viral entry. After removing the supernatants, the cells were washed three times with PBS. DMEM supplemented with 10% FBS, with or without NTZ (50.0 μM), was then added, and the cells were incubated for an additional 4 h at 37°C. The supernatants were then harvested at 26 hpi, to evaluate the viral titers as previously described (28).

### 2.9 Experiment design, cDNA library construction, and sequence data analysis

PK15 cell monolayers, achieving approximately 80% confluence in a 6-well plate, were infected with PRV-HuN-LD at a MOI of 0.1 for 2 h. Following the infection, cells were rinsed with PBS and incubated with NTZ (50 μM) or DMSO for 24 h. The cells in each group were washed with precooled PBS and total RNA was extracted utilizing Trizol reagents (Hunan Aikerui Biotechnology Co., Ltd., Changsha, China). The concentration and purity of the RNA samples were assessed using a NanoDrop spectrophotometer (Thermo Fisher, United States).

Eukaryotic mRNA was enriched using Oligo (dT) magnetic beads, and then fragmented into short segments with a fragmentation buffer. The obtained mRNA served as a template for the synthesis of first-strand cDNA utilizing random primers. Following this, a second-strand labeling buffer and a second-strand end repair enzyme mix were incorporated into the reaction system to generate second-strand cDNA. The double stranded cDNA fragments were purified with AMPure XP beads and underwent end repair. A sequencing linker was ligated to the fragments, and size selection was performed using AMPure XP beads. The cDNA library was enriched through PCR amplification. After the completion of the library construction, the library was diluted to a concentration of 1 ng/μL. The inserted size was assessed using the Agilent 2,100 Bioanalyzer (Agilent Technologies, CA, United States). RT-qPCR was performed to improve or ensure the quality of the constructed library, and the constructed library was dispatched to a commercial company (Wuhan Jinkairui Biotechnology Co., Ltd., Wuhan, China) for sequencing on the Illumina HiSeq 2,500 platform.

Raw reads that included adapter and poly-N sequences, as well as those of low-quality reads, were removed. The remaining raw reads were aligned to the genomes of the PRV variant strain (GenBank accession numbers: KP098534) and the *Sus scrofa* reference genome using the Hisat2 software. For each sequenced sample, the mapped reads were assembled using StringTie software (29).

A total of 10 core genes linked to antioxidative stress pathway, the MAPK signaling pathway, and the Wnt/*β*-catenin signaling pathway-related genes, were selected based on the results of transcription analysis to verify the differential expression of DEGs through RT-qPCR. The primers targeting these genes were shown in Supplementary Table 1.

### 2.10 Statistical analysis

All experiments conducted in this study were executed with more than three independent replicates. The data obtained from different groups were subjected to statistical analysis using a t-test, facilitated by GraphPad Prism Version 8.0 software (GraphPad Software, La Jolla, CA, United States). A *p* value of less than 0.05 was deemed statistically significant, *p < 0.05, **p < 0.01, ***p < 0.001, while ns indicates no statistical significance.

## 3 Results

### 3.1 The cytotoxicity of NTZ on PK15 and Vero cell lines

According to the findings of prior studies, we assessed the cytotoxic effects of NTZ at various concentrations on Vero and PK15 cells for 48 h utilizing the CCK-8 assay. As shown in Figures 1B,C, the highest non-toxic concentration of NTZ for PK15 cell and Vero cells was approximately 75.0 μM and 50.0 μM, respectively. The 50% cytotoxicity concentrations were determined to be 203.0 ± 3.65 μM for PK15 cells, and 217.2 ± 4.28 μM for PK15 cells.

### 3.2 NTZ treatment effectively inhibits PRV-HuN-LD infection across various cell lines

**Figure 3:**
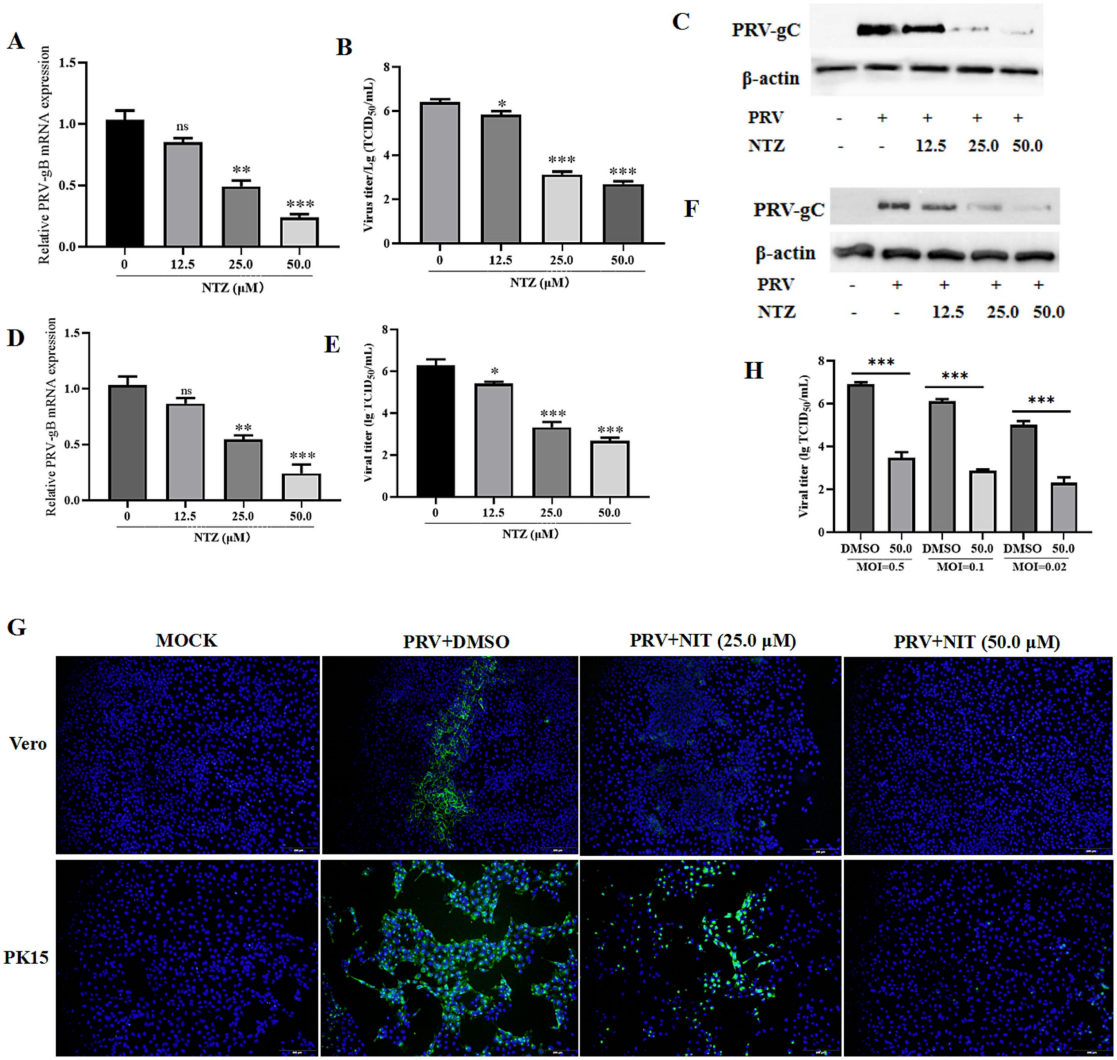
Antiviral property of NTZ against the PRV HuN-LD strain *in vitro*. **(A–C,G)** Vero cells were pre-treated with varying concentrations of NTZ (12.5, 25.0, and 50.0 μM), and then infected with PRV at a MOI of 0.1. At 24 hpi, both the cells and supernatants were collected for RT-qPCR **(A)**, TCID_50_ **(B)**, western blotting **(C)**, and IFA **(G)** assays to evaluate viral replication. **(D–F,G)** PK15 cells were pre-treated with varying concentrations of NTZ (12.5, 25.0, and 50.0 μM), and then infected with PRV at a MOI of 0.1. Following a 24-hpi period, both the cells and supernatants were harvested for RT-qPCR **(D)**, TCID_50_ **(E)**, western blotting **(F)**, and IFA **(G)** assays to evaluate viral replication. **(H)** Vero cells were pre-treated with NTZ (50.0 μM) for 1 h, and then infected with PRV at MOIs of 0.5, 0.1 or 0.02. Supernatants and cells were collected at 24 hpi to quantify viral titers.

The antiviral efficacy of NTZ against PRV-HuN-LD infection was assessed using Vero and PK15 cell lines. Each cell line was pre-treated with varying concentrations of NTZ (12.5, 25.0, and 50.0 μM), and then infected with PRV at a MOI of 0.1. Following a 24-hpi period, both the cells and supernatants were harvested for western blotting, IFA, RT-qPCR, and TCID_50_ to evaluate viral replication. The findings indicated that NTZ treatment resulted in a does-dependent reduction in the relative expression of PRV gC in Vero cells. Meanwhile, the relative mRNA expression levels of the PRV gB gene, as well as the viral titers in PRV-infected cells, were significantly higher compared to those in NTZ-treated cells (Figures 3A–C,G). Additionally, similar antiviral properties of NTZ against PRV infection were also monitored in PK15 cells (Figures 3D–F,G).

Next, Vero cells were pre-treated with NTZ (50.0 μM) for 1 h, and then infected with PRV at MOIs of 0.5, 0.1 or 0.02. Supernatants and cells were collected at 24 hpi to quantify viral titers, The results demonstrated that viral titers in the supernatants and cells from PRV-infected groups were significantly higher than those from NTZ-treated groups, which was in a manner dependent on the MOI (Figure 3H).

### 3.3 NTZ treatment demonstrates antiviral efficiency against the infections of other PRV strains and HSV-1 *in vitro*

The aforementioned results indicated NTZ effectively inhibited PRV-HuN-LD infection across Vero and PKL15 cell lines. Subsequently, additional strains, including PRV-YuN-KM (a different variant of the PRV strain), PRV-HuN-XT (a classical PRV strain), and HSV-1 strains, were employed to further investigate the antiviral efficacy of NTZ against a broad spectrum of herpesvirus infections in Vero cells. Cells were pre-treated with NTZ at concentrations of 25 or 50 μM for 2 h, followed by infection with PRV-YuN-KM, PRV-HuN-XT, or HSV-1 with an MOI of 0.1. At 24 hpi, supernatants were collected for viral titers analysis, the cells were harvested to assess the mRNA expression levels of gB gene for both PRV and HSV-1 strains. As illustrated in Supplementary Figure 1, the transcription levels of the gB genes for PRV and HSV-1 in Vero cells exhibited a significant reduction when compared to the DMSO-treated control group (Supplementary Figure 1A). Similarly, the viral titers of both PRV and HSV-1 strains in the DMSO-treated cells were markedly higher than those observed in the NTZ-treated group (Supplementary Figure 1B).

### 3.4 NTZ treatment impedes PRV replication phase

In the following study, the impact of drug addition at various time points on PRV infection was examined through western blotting and viral titer analysis (Figure 2A). The results indicated that NTZ demonstrated a significant antiviral effect against PRV infection in the post-treatment model, while no such effect was observed in the pre-treatment or co-treatment models (Figures 2B,C). The viral titer analysis revealed inhibition rates approaching 100% in the post-treatment scenario (Figure 2C). Next, we aimed to determine the specific phase of the viral life cycle-namely viral binding, entry, and release phases, at which NTZ treatment exhibits its antiviral effects against PRV infection (Figure 2D). However, no significant variations in viral titers or viral copies were monitored in the experimental models assessing viral absorption, entry, and release (Figure 2E). In conclusion, NTZ effectively suppressed PRV infection *in vitro* by targeting viral replication phase, while exhibiting no effects on other stages of the viral life cycle.

### 3.5 Transcriptomic profiling analysis of the potential cellular pathway regulated by NTZ

**Figure 4:**
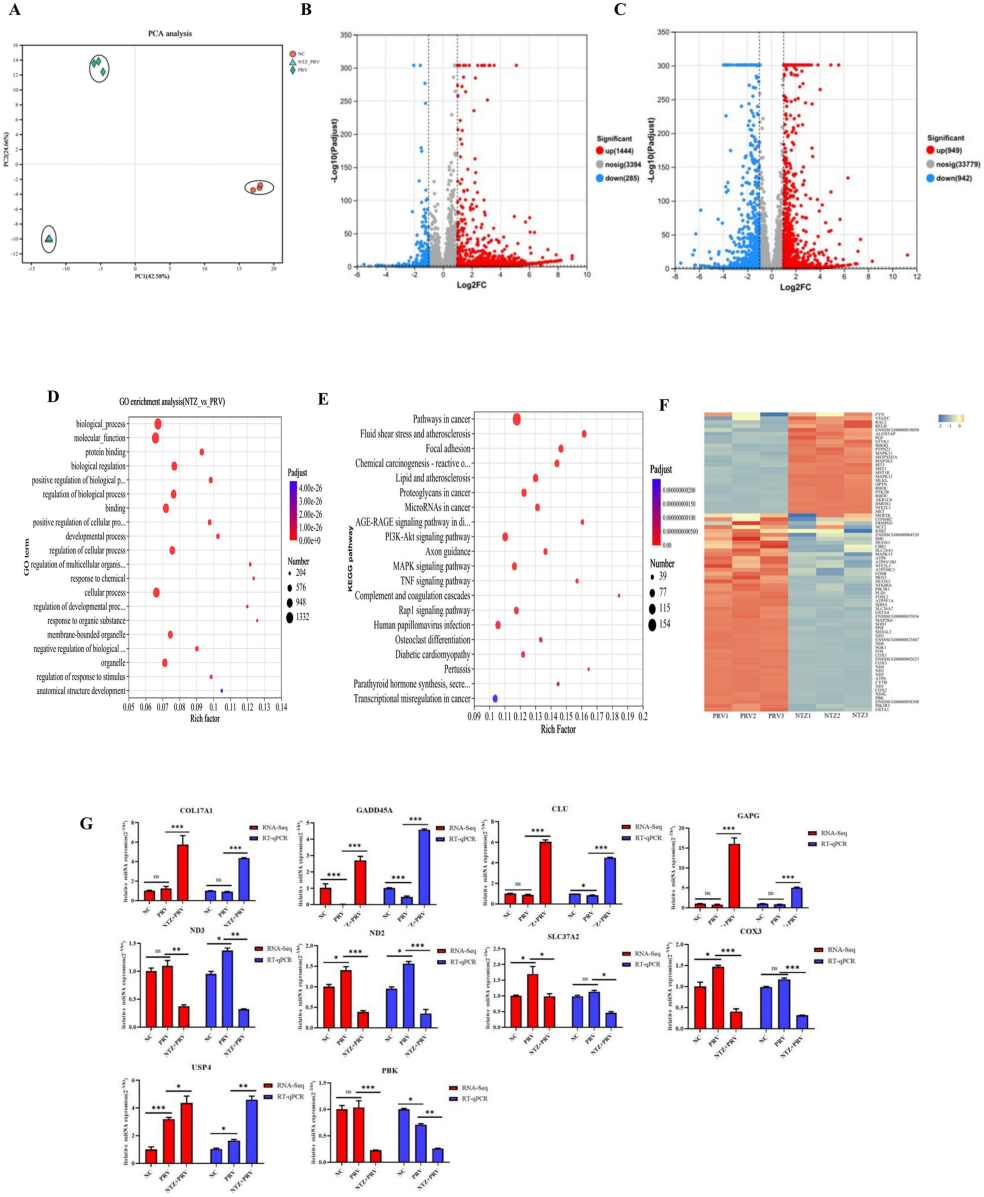
Transcriptomic profiling analysis of the potential cellular pathway regulated by NTZ. **(A)** PCA analysis of all RNA-Seq samples. **(B)** Differentially expressed genes (DEGs) identified between the mock-infected PK15 cells and PRV-infected PK15 cell, with red and green dots denoting significantly upregulated and downregulated genes, respectively. **(C)** DEGs between the mock PRV-infected PK15 cells and NTZ-treated PRV-infected PK15 cells. The red and green dots represent the significantly upregulated and downregulated genes, respectively. **(D,E)** Gene Ontology **(D)** and Kyoro Encyclopedia of Genes and Genomes **(E)** enrichment analyses of DEGs between PRV-infected PK15 cells and NTZ-treated PRV-infected PK15 cells. **(F)** Heat map analysis of the DEGs associated with the oxidative stress pathway, where red and blue colors represent upregulated and downregulated DEGs, respectively. **(G)** Validation of the mRNA expression levels of selected DEGs identified in the RNA-Seq data though RT-qPCR.

To explore the potential cellular mechanisms underlying the antiviral effects of NTZ against PRV infection, an analysis of the impact of NTZ on the host transcription in PRV-infected PK15 cells was conducted using RNA sequencing (RNA-Seq; Figure 4A). The findings revealed that a total of 1729 differentially expressed genes (DEGs) were detected in PRV-infected PK15 cells when compared to mock cells, comprising 1,444 up-regulated DEGs and 285 down-regulated DEGs (Figure 4B). In contrast, a total of 1891 DEGs were identified in PRV-infected PK15 cells relative to NTZ-treated PRV-infected cells, which included 949 up-regulated DEGs and 942 down-regulated DEGs (Figure 4C).

In terms of the DEGs between PRV-infected cells and NTZ-treated PRV-infected cells, the Kyoto Encyclopedia of Genes and Genomes (KEGG) analysis demonstrated that a significant proportion of DEGs were associated with human diseases, encompassing pathways in cancer, lipid and atherosclerosis, and chemical carciingenesis-reactive oxygen species (Figures 4D,E). The findings from the heat map analysis focused on the oxidative stress pathway revealed an up-regulation of several antioxidant genes, specifically HMOX1, NFE2L1, MAPK11, MAP3K5, MAPK12, and AKR1C8 (Figure 4F). Conversely, there was a down-regulation of certain oxidant genes, including CYTB, PIK3R3, FOS, and DUOX2, in PK15 cells infected with PRV and treated with NTZ, in comparison to the PRV-infected cells (Figure 4F).

In order to evaluate the accuracy and reliability of the RNA-Seq analysis results, the relative mRNA expression levels of genes associated with the antioxidative stress pathway (COX3, ND2, ND3, SLC37A2, and PBK), the MAPK signaling pathway (GADD45A, DSUP4), and the Wnt/*β*-catenin (USP4 and COL17A1) signaling pathway-related genes were analyzed using the RT-qPCR method. The results obtained from the RNA-Seq and RT-qPCR analyses across different groups demonstrated a high degree of consistency (Figure 4G).

## 4 Discussion

Currently, PRV continues to be regarded as a substantial threat to the swine industry in China. Despite the implementation of effective vaccines aimed at the prevention and control of PR, the prevalence of positive cases at the farm level remains notably high (16). Moreover, recent research has demonstrated that PRV may be an overlooked zoonotic pathogen, while effective strategies for the treatment and management of PRV infection are still scare (7). A variety of compounds have been shown to exhibit antiviral properties against PRV infection in both *in vivo* and *in vitro* studies, nevertheless, the number of that received approval from the Food and Drug Administration (FDA) for their efficiency against PRV is still limited (30, 31).

NTZ has been granted approved by the FDA as a safe and effective therapeutic agent for the treatment and management of parasitic infections (32). In recent years, there has been significant interest in the antiviral properties of NTZ, particularly its efficiency in inhibiting infections caused by various swine viruses (22–25). In the current study, we identified NTZ as a promising candidate antiviral agent against PRV infection. NTZ demonstrated inhibitory effects on PRV infection in both PK15 and Vero cell lines, with a CC_50_ of exceeding 200 μM and an IC_50_ of less than 12.5 μM for both cell types, resulting in a selectivity index value greater than 16. Moreover, NTZ has been shown to inhibit HSV-1 infection in Vero cells in this study, in conjunction with the fact that NTZ exhibited potential to inhibit feline herpesvirus type 1 infection *in vitro* (33), suggesting that NTZ may possess the capacity to impede infections caused by *Alphaherpesvirinae.*

The viral life cycle comprises four distinct phases: attachment, cellular entry, replication, and viral release, with host proteins playing a crucial role in these biological processes (34). NTZ has been shown to inhibit the proliferation of various viruses through diverse mechanisms. For instance, Wang *et al*. revealed that NTZ obstructs the proliferation of chikungunya virus by preventing both viral entry and cell-to-cell transmission (35). Additionally, Piacentini and his colleagues reported that NTZ mitigates the infections caused by human seasonal coronaviruses by inhibiting viral replication and interfering with the spike glycoprotein maturation (36). In the present study, we observed that the inhibitory effect of NTZ on PRV infection predominately manifests during the viral replication phase. This antiviral activity of NTZ has also been documented in other DNA viruses, including vaccinia virus (37) and canine parvovirus (38).

Natural and synthetic compounds inhibit viral infections primarily through two mechanisms: direct interaction with viral proteins (39) and indirect modulation of cellular pathways (40). RNA-Seq has emerged as a novel technology for investigating cellular transcription across various biological research domains (40–42). In this study, RNA-Seq technology was employed to elucidate the underlying antiviral mechanisms of NTZ against PRV infection in PK15 cells. The results of KEGG analysis revealed that the pathways associated with chemical carcinogenesis-reactive oxygen species, including the MAPK, P13K/Atk, and Nrf2/AKR signaling pathways, were involved in the antiviral activity of NTZ against PRV infection. Similarly, NTZ was found to significantly inhibit the replication of JEV by mitigating oxidative stress induced by the virus (25). Also, the oxidative stress pathway has been shown to play a role in the antiviral effects of several compounds against PRV infection, including Lysimachia christinae (43), polysaccharides derived from *Hippophae rhamnoides* (44), and Curcumin (45). These findings suggest that NTZ may inhibit PRV infection by reliving the oxidative stress, subsequent investigations will be undertaken to elucidate the specific pathways associated with these antiviral properties.

It is important to acknowledge that the antiviral effects of NTZ against PRV infection have not been thoroughly investigated in the present study. Firstly, the potential *in vivo* antiviral efficacy of NTZ against PRV infection remains unaddressed. Secondly, the mechanism by which NTZ may inhibit PRV infection through the targeting of viral proteins necessitates additional investigation. Furthermore, subsequent research will be undertaken to elucidate the specific cellular pathways involved in the antiviral activity of NTZ against PRV infection, as indicated by the RNA-Seq analysis.

## 5 Conclusion

In summary, the findings of this study demonstrated that NTZ significantly inhibits PRV infection by affecting the viral replication phase *in vitro*. Additionally, RNA-Seq analysis indicates that cellular signaling pathways associated with oxidative stress are involved in the antiviral efficacy of this compound against PRV infection.

## Data Availability

The original contributions presented in the study are included in the article/Supplementary material, further inquiries can be directed to the corresponding authors.

## Supplementary material

SUPPLEMENTARY FIGURE 1 NTZ broadly inhibited the infections of other PRV and HSV-1 strains. Vero cells were pre-treated with NTZ at concentrations of 25 or 50 μM for 2 hours, followed by infection with different virus strains (MOI = 0.1). At 24 hpi, supernatants were collected for viral titers analysis **(A)**, the cells were harvested to assess the mRNA expression levels of gB gene for both PRV and d HSV-1 strains **(B)**.
