# Supplementary material for: Antiviral activity of nitazoxanide against pseudorabies virus infection in vitro
Source: Front Vet Sci. 2025 Jun 16;12:1623545. doi: 10.3389/fvets.2025.1623545 (PMC12206632; doi:10.3389/fvets.2025.1623545)
Supplement: Supplementary file 1 [file Data_Sheet_1.zip › supplementary files/supplementary Table 1.docx]

**Supplementary Table 1**: primer sequences used in this study

| Gene | Sequence (3'-5') | Purpose |
| --- | --- | --- |
| q-PRV-*gB*-F | GTCCGTGAAGCGGTTCGTGAT | RT-qPCR |
| q-PRV-*gB*-R | CTCCATCATGAAGTGCGACGT |  |
| q-HSV-*gB*-F | GGACATCAAGGCGGAGAACA | RT-qPCR |
| q-HSV-*gB*-R | TTCTCCTTGAAGACCACCGC |  |
| q-*GAPDH*-F | ACCACAGTCCATGCCATCAC | RT-qPCR |
| q-*GAPDH-*R | TCCACCACCCTGTTGCTGTA |  |
| *COX3*-qPCR-F | ACCACTTACCGGAGCCCTAT | RT-qPCR |
| *COX3*-qPCR-R | ATGTGTGGTGGCCTTGGAAA |  |
| *ND2*-qPCR-F | AATCCACAGCTCAGCAACCA | RT-qPCR |
| *ND2*-qPCR-R | TTAGGCTTGTGATGACGGGT |  |
| *ND3*-qPCR-F | AACCCTAGCCTCCCTACTCG | RT-qPCR |
| *ND3*-qPCR-R | GAGGCGTGCTGATCCTATGG |  |
| *SLC37A2*-qPCR-F | TGTGGTCAAGAGTCGTCTGC | RT-qPCR |
| *SLC37A2*-qPCR-R | ATGCCGATAGCATAGGCCAC |  |
| *COL17A1*-qPCR-F | TCCTTACCACCAAAAGGGGG | RT-qPCR |
| *COL17A1*-qPCR-R | AACTGGAGGTGGAGGCATTG |  |
| *DUSP4*-qPCR-F | TTGAACGTGTCCTCCGACTG | RT-qPCR |
| *DUSP4*-qPCR-R | ACTCCTTCACCGCATCAATGT |  |
| *PBK-*qPCR-F | ATCCTTTTCCAGCAGCCGTA | RT-qPCR |
| *PBK*-qPCR-R | AGGGTCAGTCACAGTCATGT |  |
| *CLU-*qPCR-F  *CLU-*qPCR-R | CACGAGGAGAAGCAGGCG  TCGGTGGACATTTCCTGGAG | RT-qPCR |
| *CAPG-*qPCR-F | GACTCAGAGCTGCTAGCCTT | RT-qPCR |
| *CAPG-*qPCR-R | TGCTGTTTCCAGATCTCCTCC |  |
| *GADD45A-*qPCR-F | TCACTCTGATCCAGGCGTTC | RT-qPCR |
| *GADD45A-*qPCR-R | ATGTGGATTCGTCACCAGCA |  |
