# Supplementary figures and images for: Antiviral activity of nitazoxanide against pseudorabies virus infection in vitro
Source: Front Vet Sci. 2025 Jun 16;12:1623545. doi: 10.3389/fvets.2025.1623545 (PMC12206632; doi:10.3389/fvets.2025.1623545)

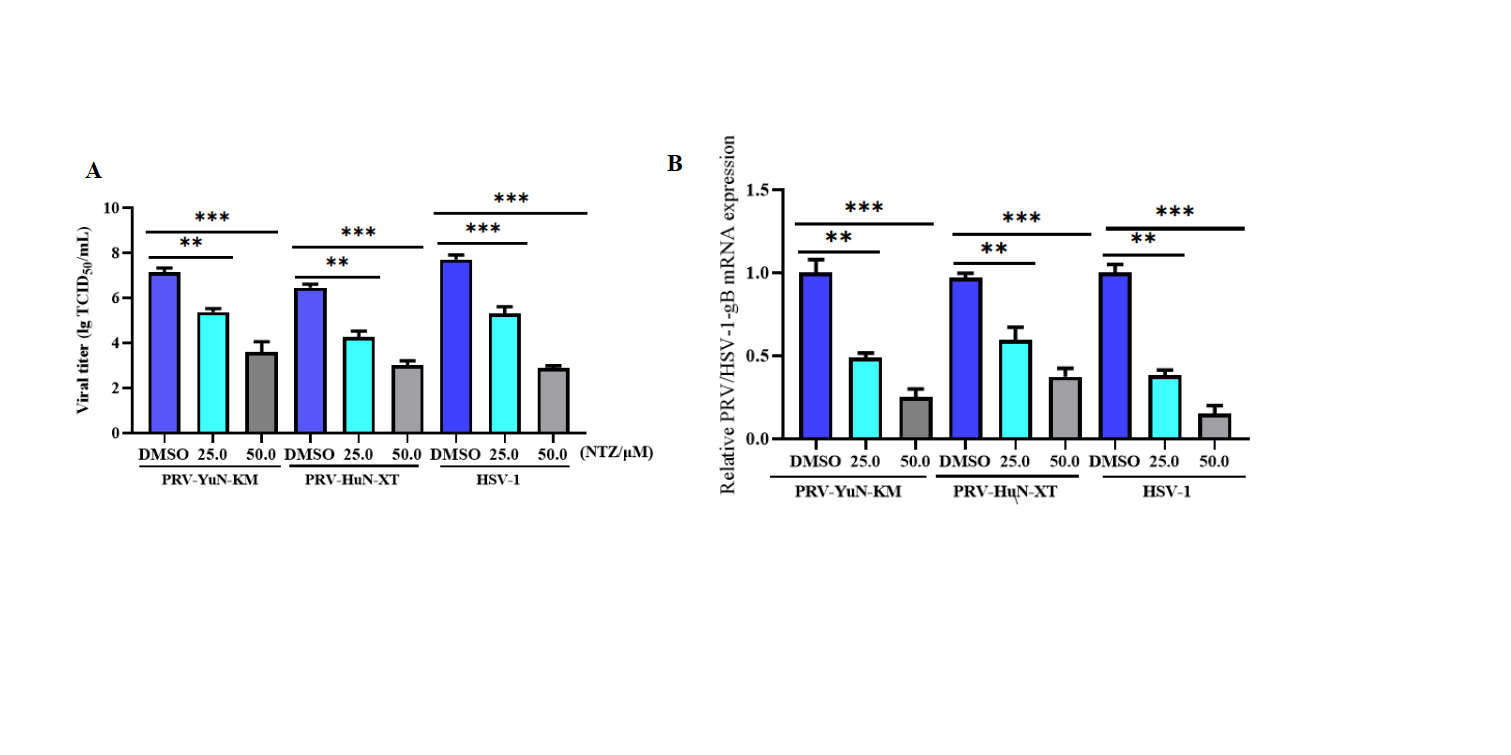

Supplement: Supplementary file 1 [file Data_Sheet_1.zip › supplementary files/Supplementary Figure 1.tif]
